# The Effect of Vitamin C on Pathological Parameters and Survival Duration of Critically Ill Coronavirus Disease 2019 Patients: A Randomized Clinical Trial

**DOI:** 10.3389/fimmu.2021.717816

**Published:** 2021-12-15

**Authors:** Nazanin Majidi, Faezeh Rabbani, Somayeh Gholami, Maryam Gholamalizadeh, Fatemeh BourBour, Samira Rastgoo, Azadeh Hajipour, Mahdi Shadnoosh, Mohammad Esmail Akbari, Bojlul Bahar, Narjes Ashoori, Atiyeh Alizadeh, Forough Samipoor, Alireza Moslem, Saeid Doaei, Katsuhiko Suzuki

**Affiliations:** ^1^ Department of Nutrition, Science and Research Branch, Islamic Azad University, Tehran, Iran; ^2^ Department of Medicinal Chemistry, Faculty of Pharmacy and Pharmaceutical Sciences Research Center, Shahid Sadoughi University of Medical Sciences, Yazd, Iran; ^3^ Razi Hospital, Guilan University of Medical Sciences, Rasht, Iran; ^4^ Cancer Research Center, Shahid Beheshti University of Medical Sciences, Tehran, Iran; ^5^ Department of Clinical Nutrition and Dietetics, Research Institute Shahid Beheshti University of Medical Science, Tehran, Iran; ^6^ School of Health, Qazvin University of Medical Sciences, Qazvin, Iran; ^7^ Department of Clinical Nutrition, Faculty of Nutrition Sciences and Food Technology, National Nutrition and Food Technology Research Institute, Shahid Beheshti University of Medical Sciences, Tehran, Iran; ^8^ Nutrition Sciences and Applied Food Safety Studies, Research Centre for Global Development, School of Sport & Health Sciences, University of Central Lancashire, Preston, United Kingdom; ^9^ Department of Pharmacognosy, Faculty of Pharmacy, Tehran University of Medical Science, Tehran, Iran; ^10^ Department of Anesthesiology, Sabzevar University of Medical Sciences, Sabzevar, Iran; ^11^ Reproductive Health Research Center, Department of Obstetrics and Gynecology, School of Medicine, Al-Zahra Hospital, Guilan University of Medical Sciences, Rasht, Iran; ^12^ Faculty of Sport Sciences, Waseda University, Tokorozawa, Japan

**Keywords:** COVID-19, vitamin C, critically ill patients, potassium, supplementation, survival

## Abstract

**Introduction:**

Vitamin C has been reported to have beneficial effects on patients with coronavirus disease 2019 (COVID-19). This study aimed to investigate the effect of vitamin C supplementation on pathological parameters and survival duration of critically ill patients with COVID-19.

**Methods:**

This clinical trial was conducted on 120 hospitalized critically ill patients infected with COVID-19. The intervention group (n = 31) received one capsule of 500 mg of vitamin C daily for 14 days. The control group (n = 69) received the same nutrition except for vitamin C supplements. Measurement of pathological and biochemical parameters was performed at baseline and after 2 weeks of the intervention.

**Results:**

Following 2 weeks of vitamin C supplementation, the level of serum K was significantly lower in the patients compared with the control group (3.93 vs. 4.21 mEq/L, *p* < 0.01). Vitamin C supplementation resulted in a higher mean survival duration compared with that of the control group (8 vs. 4 days, *p* < 0.01). There was a linear association between the number of days of vitamin C intake and survival duration (B = 1.66, *p* < 0.001). The vitamin C supplementation had no effect on blood glucose, mean arterial pressure, arterial blood gas (ABG) parameters, Glasgow Coma Scale (GCS), kidney function, cell blood count (CBC), hemoglobin (Hb), platelet (Plt), partial thromboplastin time (PTT), albumin, hematocrit (Hct), and other serum electrolytes including sodium (Na), calcium, and phosphorus (P).

**Conclusion:**

The present study demonstrated the potential of vitamin C supplementation in enhancing the survival duration of critically ill patients with COVID-19.

**Clinical Trial Registration:**

https://www.irct.ir/trial/55074, identifier IRCT20151226025699N5

## Introduction

The coronavirus disease 2019 (COVID-19) pandemic is the most important global health crisis of recent years. Caused by a highly contagious virus, the infection led to a large number of deaths and widespread contamination throughout the world (1). By November 2020, the severe acute respiratory syndrome coronavirus-2 (SARS-CoV-2) caused more than 50 million confirmed cases of COVID-19, including about 1.5 million deaths worldwide (2). At the same time in Iran, more than 700,000 confirmed cases of COVID-19 and more than 40,000 deaths were reported (3).

The most common symptoms of COVID-19 include fever, cough, fatigue, and myalgia. However, the disease progresses in some cases, and patients experience dyspnea, respiratory dysfunctions, shock, heart disease, hypertension, and multiple organ damage, which may finally lead to death (4). Higher age, obesity, weakened immune system, and underlying diseases such as diabetes are the known risk factors associated with disease severity (5).

Micronutrients play crucial roles in supporting the immune system during viral infection (6). Ascorbic acid or vitamin C was reported to be necessary for the proper function of the immune system. During acute viral infections, serum and leukocyte levels of vitamin C decreases, and the patients’ requirement for vitamin C increases along with the progression of the infection (7, 8). It is reported that high doses of vitamin C might have an antiviral effect through the inactivation of viral multiplication, thereby reducing the viral load (8). Previous studies have reported that vitamin C inhibited replication of some viruses such as herpes simplex virus, poliovirus, and influenza (7, 9). A recent study reported that a high-dose intravenous vitamin C had positive effects in the treatment of patients with moderate to severe COVID-19 (10). However, promising results were obtained when vitamin C was administered to the patients (11, 12), and the beneficial effects of vitamin C administration in critically ill patients with the COVID-19 infection remained controversial. So this study aimed to evaluate the effect of vitamin C supplementation on the biochemical and pathological parameters and survival duration in critically ill patients with COVID-19.

## Methods

### Study Design and Population

**Figure 1:**
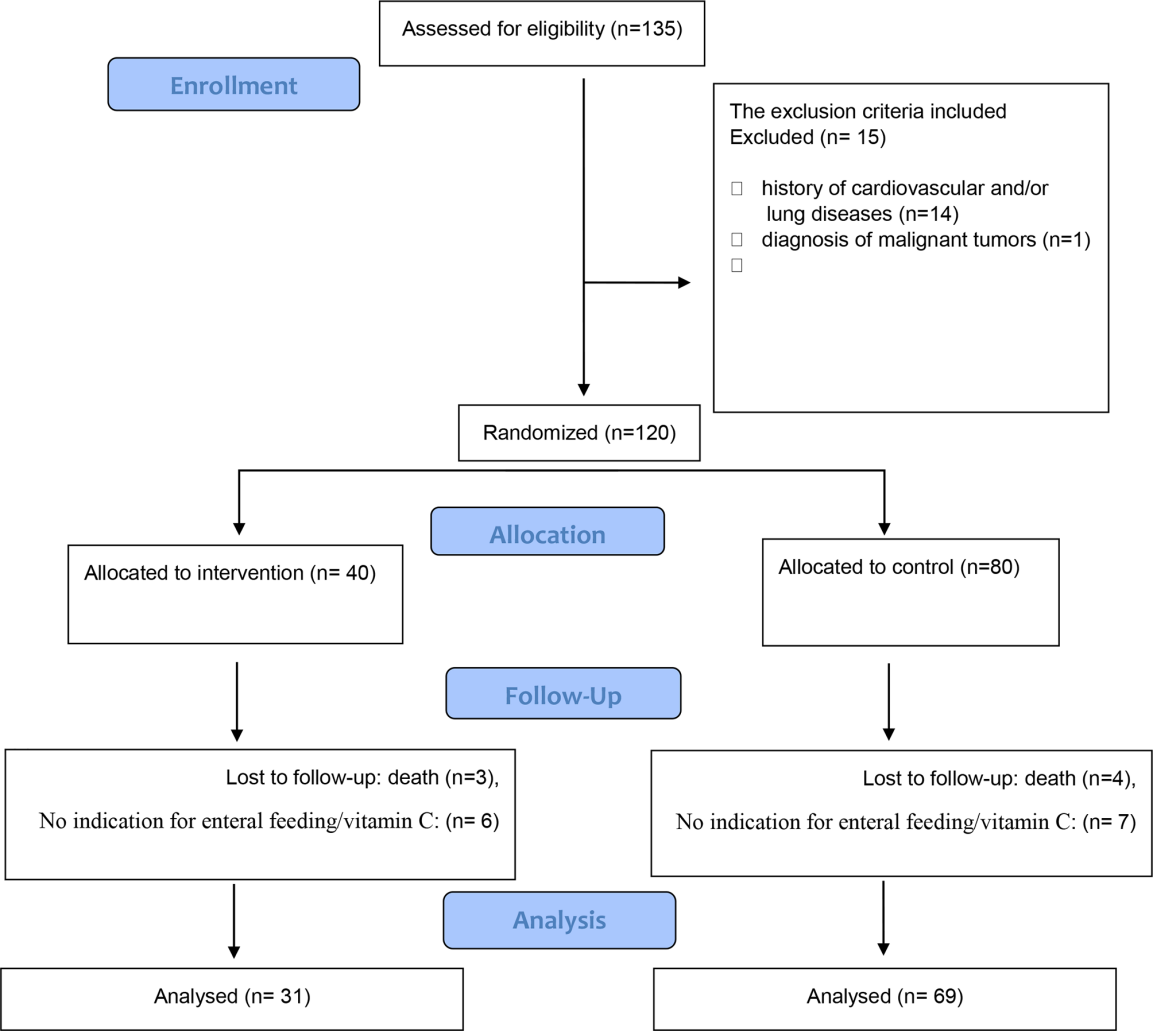
Study flowchart.

This study was a double-blind, randomized clinical trial carried out from May to July 2020 on critically ill patients infected with SARS-CoV-2 in Razi Hospital, Rasht, Iran. The inclusion criteria were the age between 35 and 75 years, diagnosed as COVID-19 positive, patients likely to be in the intensive care unit (ICU) for at least 48 h, and have an indication for enteral nutrition. The main outcome was the survival duration of the patients, and the secondary outcomes were clinical and biochemical measurements. A total of one hundred thirty-five patients were initially assessed for eligibility and met the inclusion criteria, where α = 0.95, β = 20%, and study power = 80%. The 2:1 ratio of control to intervention sample was used because of a fixed and limited budget for this research project. Therefore, more participants were randomized to the cheaper arm in order to facilitate greater overall recruitment in the face of a possibly high dropout rate (13). The non-inclusion criteria included the history of cardiovascular and/or lung diseases (n = 14), diagnosis of malignant tumors (n = 1) because of their effects on current medical treatment and diet, and intake of vitamin C supplements during the prior 3 months before the study. The participants were assigned randomly to the case (n = 40) and control (n = 80) groups. The exclusion criteria included no completion of the study because of death (n = 7), contraindication to enteral nutrition or intolerance to enteral nutrition so that supplementation with vitamin C is not possible (n = 7), or contraindications to vitamin C supplementation due to different reasons (such as the interaction with drugs) before 14 days of the baseline sampling (n = 6). The final analysis was performed on 100 critically ill patients infected with COVID-19, including 31 patients in the vitamin C supplementation group and 69 patients in the control group. A schematic flowchart of the study is presented in **Figure 1**.

### Interventions

This study was done as a double-blind, without placebo trial, such that the patients and researchers were not aware of the arms of the study. The allocation to the groups was done through web-based randomization using https://www.randomizer.org. Sealed non-transparent envelopes with randomized sequences were used to hide the allocation. Vitamin C supplementation was done by a nurse who was not a member of the research team. Also, the required information about the desired outcomes was collected by another nurse. Supplementation was started after 24 h of hospitalization in the ICU after the stable hemodynamic condition of the patient was confirmed by the treating physician. Finally, the results were analyzed by a person outside of the treatment team. Due to the reported side effects of high-dose vitamin C intravenously (12), in this study, low-dose vitamin C was administered through enteral nutrition. The intervention group received one capsule of 500 mg of vitamin C daily (Vita Pharmed, Switzerland) by adding the supplement to their enteral feeding. The control group received the same nutritional support using the same route, although no vitamin C was added to their enteral formula.

### Data Collection

After the written consent forms were collected, the required information was collected using a predetermined checklist including the following data.

#### Medical History

Data on previous illnesses, medications, blood pressure, random blood glucose, respiratory status, and defecation were collected using medical records.

#### Anthropometric and Dietary Assessments

The length of the ulna was measured, while the forearm was flexed at the elbow from the tip of the olecranon process to the tip of the styloid process with a centimeter tapeline with an accuracy of 0.5 cm. The actual weight was then estimated using the standard charts. The body mass index (BMI) was calculated as body weight/height^2^ (kg/m^2^). Data on daily intake of enteral nutrition, type of enteral formula (high protein, diabetic, or standard), the amount of caloric intake, and vitamin and mineral supplements were collected using data recorded in ICU sheets.

#### Biochemical and Clinical Indices

Measurements of biochemical indices were routinely performed in the hospital using standard kits, and the required data were gathered from the lab test section of the ICU sheets. Data on the level of white blood cell count (including neutrophil, lymphocyte, and monocyte), hemoglobin (Hb), platelet (Plt), the partial thromboplastin time (PTT), albumin, and hematocrit (Hct), blood glucose, erythrocyte sedimentation rate (ESR), kidney function parameters (i.e., blood urea nitrogen (BUN), creatinine (Cr), and urine volume), and serum electrolytes (i.e., sodium (Na), potassium (K), calcium, and phosphorus (P)) were collected at baseline and after 14 days of the intervention.

In terms of clinical indices, the level of arterial blood gas (ABG) parameters (i.e., O_2_ saturation, arterial pH, partial pressure of oxygen (PO_2_), partial pressure of carbon dioxide (PCO_2_), bicarbonate (HCO_3_), and base excess (Be)), mean arterial pressure (MAP), and Glasgow Coma Scale (GCS) were measured at baseline and after the intervention. Moreover, the survival duration in ICU after the study was assessed on the 14th day after the end of the study, which refers to the number of survival days that patients survived in the ICU.

### Statistical Analyses

The quantitative data were presented as the mean ± SD, and the qualitative data were presented as the numbers and percentages to describe the status of demographic, social, and anthropometric measurements of the participants. The Kolmogorov–Smirnov test was used to verify the normal distribution of the results of each variable. Independent t-test and chi-square test were used to compare the results between the groups at baseline. The general linear model repeated measure was used to identify the effect of vitamin C supplementation on inflammatory and biochemical markers and to compare the values in the intervention and control groups after adjusting for the confounding factors including age, sex, BMI, dietary intake, smoking, presence of background diseases such as diabetes and hypertension, and medicines and supplements. All statistical analyses were performed using the SPPS package version 21.0 (Statistical Product and Service Solutions, Chicago, IL, USA). *p*-Values of less than 0.05 were considered statistically significant.

## Results

The general characteristics of the patients included in this study are presented in **Table 1**. All the patients received high protein enteral formula. No difference was found on age, weight, height, daily formula intake, BMI, Acute Physiologic and Chronic Health Evaluation (APACHE II), gender, underlying disease, and addiction between the groups.

**Table 1:** General characteristics (at baseline) of the patients in the vitamin C supplementation and control groups.

Parameters	Vitamin C supplementation (n = 31)	Control (n = 69)	*p*-Value
Age (years)	59.42 ( ± 15.07)	63.82 ( ± 14.58)	0.170
Males	19 (61%)	41 (58%)	0.830
Weight (kg)	76.33 ( ± 19.12)	75.51 ( ± 8.86)	0.770
Height (cm)	166 ( ± 6.63)	166 ( ± 6.73)	0.630
BMI (kg/m^2^)	27.53 ( ± 7.15)	27.45 ( ± 3.32)	0.930
Having underlying disease*	14 (45%)	28 (41%)	0.760
APACHE II	15.50 ( ± 1.69)	15.43 ( ± 1.93)	0.880
Addiction	1 (3%)	6 (9%)	0.420
Daily formula intake (cm^3^)	1,051 ( ± 579.58)	930 ( ± 341.07)	0.101

BMI, body mass index; APACHE II, Acute Physiologic and Chronic Health Evaluation.

^*^Diabetes, chronic kidney disease, and hypertension.

The clinical and pathological biomarkers assessed in the vitamin C supplementation and control groups at baseline and post-intervention are presented in **Table 2**. Of the serum electrolytes evaluated, the level of serum K remained unchanged in the vitamin C supplementation group (3.92 vs. 3.93 mEq/L). However, there was a significant (*p* < 0.05) increase of the serum K levels between the baseline (3.75 mEq/L) and post-intervention (4.21 mEq/L) in the control group. Nevertheless, the serum K level in both groups remained within the normal physiological range. For the other serum electrolytes (Na, Ca, and P) measured, no significant difference between the vitamin C supplementation and control groups was evident after intervention (**Table 2**). Vitamin C supplementation had no effect on the parameters of kidney function including the levels of BUN, Cr, and excreted urine (**Table 2**). Furthermore, no significant difference was found between the levels of ABG parameters including arterial pH, HCO_3_, Be, PO_2_, and PCO_2_ between the two groups as was the case for GCS.

**Table 2:** Clinical and pathological biomarkers at pre- and post-intervention in vitamin C supplementation and control groups.

Biomarkers	Vitamin C group (n = 31)		Control group (n = 69)		F	*p*-Value*
	Pre-intervention	Post-intervention	Pre-intervention	Post-intervention		
Blood sugar (mg/dl)	147 ( ± 55.42)	148 ( ± 53.22)	148 ( ± 48.83)	153 ( ± 45.23)	0.08	0.780
INR	1.053 ( ± 0.12)	1.07 ( ± 0.17)	1.03 ( ± 0.32)	1.023 ( ± 0.40)	0.08	0.780
PTT (s)	40.54 ( ± 8.09)	42.92 ( ± 7.77)	40.28 ( ± 6.21)	40.14 ( ± 7.90)	1.41	0.240
PT (s)	12.51 ( ± 1.08)	13.01 ( ± 1.94)	12.71 ( ± 1.07)	12.45 ( ± 0.80)	0.47	0.490
Plt	2.29 ( ± 96.87)	1.78 ( ± 83.08)	2.05 ( ± 78.67)	1.90 ( ± 57.76)	0.15	0.700
Hb (g/dl)	10.40 ( ± 1.15)	9.37 ( ± 1.25)	9.8 ( ± 1.21)	9.10 ( ± 1.56)	0.23	0.630
GCS	8.15 ( ± 0.92)	7.84 ( ± 0.92)	8.57 ( ± 0.97)	8.28 ( ± 0.75)	1.33	0.260
Lymphocyte (10^6^/L)	1,175 ( ± 240)	1,175 ( ± 358)	1,075 ( ± 250)	925 ( ± 427)	0.002	0.960
Neutrophil (10^6^/L)	8,620 ( ± 377)	8,660 ( ± 459)	8,800 ( ± 141)	8,950 ( ± 238)	0.003	0.960
WBC (10^6^/L)	11,477 ( ± 405)	11,828 ( ± 291)	10,389 ( ± 131)	13,785 ( ± 378)	1.20	0.284
Be (mEq/L)	−4.44 ( ± 6.63)	−4.72 ( ± 7.67)	−4.22 ( ± 5.83)	−3.82 ( ± 5.55)	2.51	0.127
HCO_3_ (mEq/L)	21.68 ( ± 7.08)	21.87 ( ± 7.78)	22.68 ( ± 7.89)	21.5 ( ± 6.16)	0.00	0.990
PCO_2_ (mmHg)	45.90 ( ± 16.93)	44.35 ( ± 12.48)	42.02 ( ± 13.78)	40.70 ( ± 8.90)	0.003	0.959
PO_2_ (mmHg)	67.45 ( ± 23.10)	67.84 ( ± 23.02)	71.68 ( ± 3.87)	72.20 ( ± 8.21)	0.00	0.980
Arterial pH	7.27 ( ± 0.08)	7.28 ( ± 0.07)	7.25 ( ± 0.79)	7.27 ( ± 0.50)	0.797	0.382
O_2_ sat.	84.54 ( ± 8.89)	83.21 ( ± 7.52)	82.00 ( ± 6.73)	84.14 ( ± 3.71)	0.076	0.780
MAP	73.36 ( ± 9.53)	74.28 ( ± 10.43)	72.14 ( ± 2.60)	73.14 ( ± 3.23)	0.001	0.980
P (mg/dl)	3.19 ( ± 1.22)	3.27 ( ± 1.15)	3.30 ( ± 1.24)	2.94 ( ± 0.60)	0.21	0.880
Ca (mg/dl)	8.05 ( ± 0.66)	7.85 ( ± 0.48)	7.55 ( ± 0.07)	7.75 ( ± 0.35)	1.009	0.490
Hct (%)	31.83 ( ± 3.40)	29.19 ( ± 3.58)	30.88 ( ± 3.01)	29.40 ( ± 4.60)	0.009	0.927
Alb (g/dl)	3.03 ( ± 0.35)	2.95 ( ± 0.29)	2.80 ( ± 0.00)	2.90 ( ± 0.14)	0.132	0.750
Cr (mg/ml)	1.35 ( ± 1.27)	1.36 ( ± 0.90)	1.38 ( ± 1.4)	1.25 ( ± 0.89)	0.05	0.820
BUN (mg/ml)	38.40 ( ± 24.88)	34.52 ( ± 20.04)	35.57 ( ± 22.61)	31.42 ( ± 15.20)	0.20	0.650
K (mEq/L)	3.92 ( ± 0.51)	3.93 ( ± 0.35)	3.75 ( ± 0.25)	4.21 ( ± 0.46)	7.67	0.010
Na (mEq/L)	1.38 ( ± 5.75)	1.39 ( ± 5.15)	1.37 ( ± 3.72)	1.38 ( ± 3.56)	0.04	0.840
ESR (mm/h)	87.00 ( ± 1.41)	87.00 ( ± 1.41)	90.50 ( ± 2.12)	90.50 ( ± 2.12)	0.1	0.100
Urine volume (L)	1.83 ( ± 620.08)	2.11 ( ± 848.09)	2.04 ( ± 710.88)	1.85 ( ± 316.79)	0.56	0.460

Values within the parentheses are SDs of means.

Na, sodium; K, potassium; BUN, blood urea nitrogen; Cr, creatinine; Alb, albumin; Hct, hematocrit; Ca, calcium; P, phosphorus; MAP, mean arterial pressure; O_2_ sat., oxygen saturation; pH, potential hydrogen; PO_2_, partial pressure of oxygen; PCO_2_, partial pressure of carbon dioxide; HCO_3_, bicarbonate; Be, base excess; WBC, white blood Cell; GCS, Glasgow Coma Scale; Hb, hemoglobin; Plt, platelet; PTT, partial thromboplastin time (test); PT, prothrombin time (test); ESR, erythrocyte sedimentation rate; INR, international normalized ratio of prothrombin time of blood coagulation.

*General linear model for repeated measures for the interaction of time and group corrected for the baseline variable.

For the blood biomarkers, no significant differences were found in complete blood count (CBC), levels of lymphocyte, PTT, PT, Hct, neutrophil, monocyte, Hb, ESR, international normalized ratio (INR), and Plt between the vitamin C supplementation and control groups post-intervention (**Table 2**). No significant differences were also evident in the other factors including blood glucose, albumin, MAP, and O_2_ saturation.

**Figure 2:**
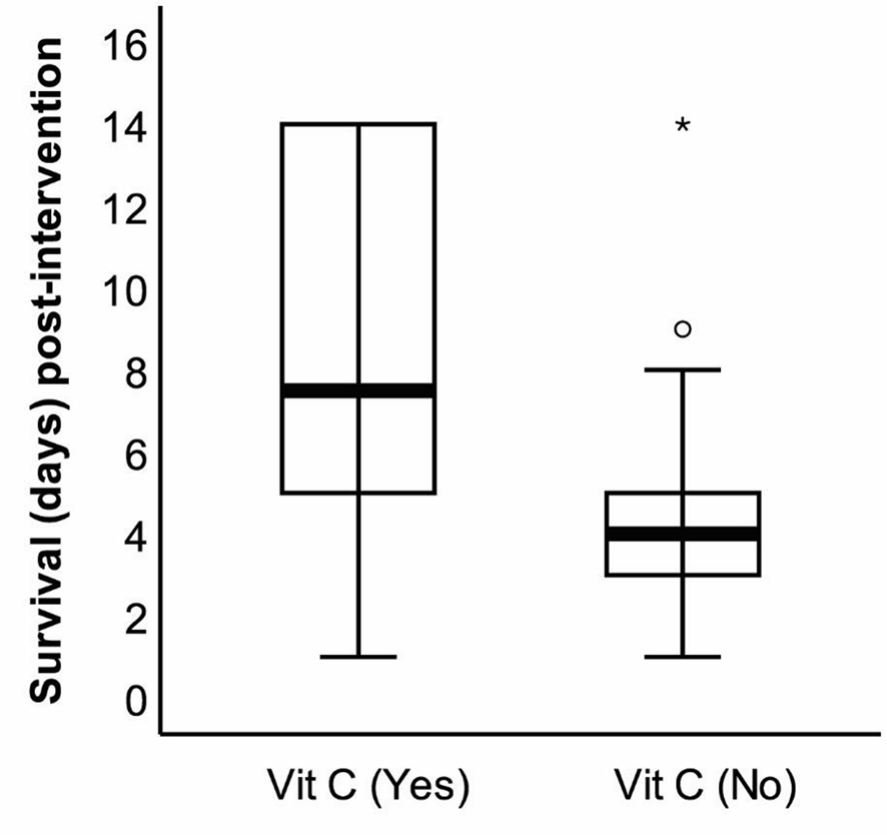
The survival duration of the patients with coronavirus disease 2019 (COVID-19) on the 14th day after the end of the study. Following the intervention, survival duration was recorded on the 14th day (28th day since the beginning of the study), and the difference was statistically significant (*p < 0.01).

The effect of vitamin C supplementation on the survival of the patients was assessed on the 14th day following the completion of the intervention trial. The survival duration significantly increased by the supplementation as evident on the 14th day post-intervention (**Figure 2**). Following 2 weeks of completion of the intervention trial, a significantly (*p* = 0.028) higher number of patients in the intervention group (16.1%) compared with the control group (2.9%) survived. The regression analysis further revealed that the survival duration of the patients had a linear positive association with the duration of vitamin C supplementation (B = 1.66, *p* = 0.001). This positive association did not alter even after adjusting for age and BMI (B = 1.59, *p* = 0.001) (model 2) and after further adjustments for APACHE II, underlying diseases, use of ventilator, and nutrition therapy (B = 1.27, *p* = 0.001) (model 3) (**Table 3**).

**Table 3:** The linear association of the number of days of vitamin C supplementation (500 mg/day) and survival duration.

	Model 1		Model 2		Model 3	
	B	*p*	B	*p*	B	p*
Survival duration in ICU (day)^*^	1.66	0.001	1.59	0.001	1.27	0.001

Model 1: crude. Model 2: adjusted for age and BMI. Model 3: further adjustments for APACHE II, underlying diseases, use of a ventilator, and nutrition therapy.

ICU, intensive care unit; APACHE II, Acute Physiologic and Chronic Health Evaluation; BMI, body mass index.

*The number of survival days that patients survived in the ICU.

## Discussion

The present study demonstrated that vitamin C supplementation for 2 weeks significantly increased the survival duration of the COVID-19 patients during the post-supplementation period. The results of this study further indicated that the vitamin C supplementation had no adverse effect on the kidney function, ABG parameters, GCS, CBC, and other serum electrolytes including the levels of Na, Ca, and P.

The benefits of vitamin C supplementation in viral infection, including COVID-19, are reported in the literature. A recent study reported that administration of a high dose of intravenous vitamin C (HDIVC) led to a shorter mean hospital length of stay (LOS) in patients with COVID-19 and concluded that HDIVC may provide a protective clinical effect in critically ill patients with COVID-19 (14). While a low serum level of vitamin C was reported in viral infections, the use of a high dosage >1,000 mg to boost the serum level was reported in the literature. However, when a high dose of vitamin C was administered, it was used only for a shorter time, generally for <5 days (15). In healthy volunteers, a daily dose of >500 mg tends to reduce the bioavailability of vitamin C. Any excess amount of vitamin C administered is likely to be excreted out. Hence, a dose beyond 500 mg has no evident value (16). To avoid any adverse side effects of vitamin C in COVID-19 patients, a daily dose of 500 mg administered for 14 days was investigated in this study. The present study indeed demonstrated the beneficial effects of vitamin C in patients with COVID-19 even at the lower dosage (500 mg/day).

The CITRIS-ALI trial done by Fowler et al. reported that vitamin C (50 mg/kg) supplementation through intravenous route every 6 h for 96 h had no effect on disease severity scores, C-reactive protein levels, or thrombomodulin levels in patients with sepsis and acute respiratory distress syndrome (ARDS) as compared with the placebo. However, 28-day all-cause mortality was lowered with vitamin C administration, which was in line with the results of the present study (17). Another study by Tomoko et al. on patients with septic shock compared the treatment with intravenous vitamin C, hydrocortisone, and thiamine (HAT therapy) with intravenous hydrocortisone alone and reported that vitamin C did not significantly improve the duration of survival and free of vasopressor administration over 7 days (18). However, a high dosage of intravenous vitamin C in a short period was used in this study, and the possible individual effects of vitamin C were not assessed separately.

The electrolyte imbalances can frequently occur in acute systemic diseases. For example, the SARS-CoV-2 can induce hyperkalemia through influence on the renin–angiotensin–aldosterone pathway. Of the serum electrolyte markers evaluated in the present study, no major alteration in the serum electrolyte due to vitamin C supplementation was evident. The level of serum K marginally changed during the intervention period. The serum K was significantly higher in the control group than the vitamin C intervention group relative to their baseline levels. It may be likely that vitamin C supplementation helped keep the serum K level unchanged during the intervention period, while there was a marginal but significant increase in the serum K level in the control group. Vitamin C was reported to induce hypokalemia in terminally ill patients (16). Nonetheless, the serum K levels in all the patients (intervention and control) remain within the physiologically relevant range. A recent study suggested that serum K level beyond 5.0 mmol/L increased the risk of 30-day mortality in COVID-19 patients, suggesting increased serum K levels influencing the disease outcome (19). In clear contrast, Lippi et al. reported that COVID-19 severity is associated with lower serum sodium, potassium, and calcium levels (17).

The underlying mechanisms of the effect of vitamin C on COVID-19 are not clear. Previous research indicated that cytokine storms may be one of the mechanisms of the effects of highly pathogenic human coronavirus such as severe acute respiratory syndrome (20). Liu et al. reported that HDIVC could suppress cytokine storms caused by COVID-19, help improve pulmonary function, and reduce the risk of ARDS in patients with COVID-19 (21). Another mechanism that explains the role of vitamin C in COVID-19 infection is that vitamin C may help neutralize the pro-inflammatory response and combat the elevated levels of reactive oxygen species, thus limiting collateral tissue damage that frequently occurs in viral infections (22).

Vitamin C could possibly improve the treatment of patients with COVID-19 by enhancing the immunological response against viral infections and the direct effect against viral replication (7, 8). It also suppresses cytokine storm through its antioxidant and anti-inflammatory effects. In addition, vitamin C increases interferon production and stimulates lymphocyte proliferation, thereby improving the host antiviral immune response (18). The present study had some limitations. First, we did not measure serum vitamin C levels. Therefore, the condition of the patients in terms of vitamin C was not adjusted before the study. Second, due to the critical condition of the patients and uncertainty about the safety of high doses of vitamin C in these patients, the dose of vitamin C used in this study was low. Higher doses of vitamin C may have different effects. Third, although vitamin C supplementation reduced serum potassium levels of the patients with COVID-19, serum potassium levels were within the normal range in both groups. Therefore, the effect of vitamin C on serum potassium levels in this study was not clinically significant. Fourth, data on the survival rate of patients were collected only once, 14 days after the intervention, and data on the LOS and ventilator-free days (VFDs) were also not collected. Fifth, in the present study, serum levels of inflammatory and immunological factors were not measured. Therefore, the mechanism of action of vitamin C is not provable based on the present results. While the present study demonstrated the potential effect of vitamin C on the survival of COVID-19 patients, further studies with longer duration and different doses of vitamin C are warranted.

## Conclusion

The results of this study indicated a significant negative correlation between vitamin C supplementation with the level of serum K in patients with COVID-19. Daily supplementation of 500 mg vitamin C resulted in an increase in the survival duration of the patients. Our results further indicated that vitamin C supplementation had no effect on kidney function, ABG parameters, GCS, CBC, and other serum electrolytes such as Na, Ca, and P. Further clinical studies are needed to confirm the effect of vitamin C and on COVID-19.

## Data Availability Statement

The raw data supporting the conclusions of this article will be made available by the authors, without undue reservation.

## Ethics Statement

The studies involving human participants were reviewed and approved by Ethical code: IR.MEDSAB.REC.1399.195. The patients/participants provided their written informed consent to participate in this study.

## Author Contributions

NM, AH, SD, SG, SR, MG, FB, and NA designed the study and were involved in the data collection, analysis, and drafting of the manuscript. MA, BB, NA, AA, MSh, FS, AM, and KS were involved in the design of the study and analysis of the data and critically reviewed the manuscript. BB contributed to the data analysis and review of the manuscript. All authors contributed to the article and approved the submitted version.

## Funding

The funding for this study was provided by Sabzevar University of Medical Sciences, Sabzevar, Iran (code 99213).

## Conflict of Interest

The authors declare that the research was conducted in the absence of any commercial or financial relationships that could be construed as a potential conflict of interest.

## Publisher’s Note

All claims expressed in this article are solely those of the authors and do not necessarily represent those of their affiliated organizations, or those of the publisher, the editors and the reviewers. Any product that may be evaluated in this article, or claim that may be made by its manufacturer, is not guaranteed or endorsed by the publisher.

## Abbreviations

n−3 PUFAs, omega-3 polyunsaturated fatty acids; ABG, arterial blood gas; CBC, complete blood count; GCS, Glasgow Coma Scale; PO_2_, partial pressure of oxygen; PCO_2_, partial pressure of carbon dioxide; HCO_3_, bicarbonate; Be, base excess; BUN, blood urea nitrogen; Cr, creatinine.
